# Characterization of hepatitis B and delta coinfection in Israel

**DOI:** 10.1186/s12879-018-3008-x

**Published:** 2018-02-27

**Authors:** Rachel Shirazi, Daniela Ram, Aviya Rakovsky, Efrat Bucris, Yael Gozlan, Yaniv Lustig, Pninit Shaked-Mishan, Orit Picard, Yonat Shemer-Avni, Haim Ben-Zvi, Ora Halutz, Yoav Lurie, Ella Veizman, Matthias Carlebach, Marius Braun, Michal Cohen- Naftaly, Amir Shlomai, Rifaat Safadi, Ella Mendelson, Ella H. Sklan, Ziv Ben-Ari, Orna Mor

**Affiliations:** 1 Central Virology Laboratory, Ministry of Health, Chaim Sheba Medical Center, Tel - Hashomer, 52621 Ramat Gan, Israel; 2 Microbiology Laboratory, Carmel Medical Center, Haifa, Israel; 3 Gastroenterology Laboratory, Sheba Medical Center, Ramat Gan, Israel; 4 Laboratory of Clinical Virology, Soroka University Medical Center, Beer Sheva, Israel; 5 Microbiology Laboratory, Rabin Medical Center, Petach Tikva, Israel; 6 Microbiology Laboratory, Sorasky Medical Center, Tel Aviv, Israel; 7 Liver Unit, Shaare Zedek Medical Center, Jerusalem, Israel; 8 Liver Unit, Rambam Medical Center, Haifa, Israel; 9 Liver Unit, Bnai Zion Medical Center, Haifa, Israel; 10 Liver Institute, Rabin Medical Center, Petah-Tikva, Israel; 11 The Sackler School of Medicine, Tel Aviv University, Tel Aviv, Israel; 12 Liver Unit, Hadassah Hebrew University Hospital, Jerusalem, Israel; 13 School of Public Health, Sackler School of Medicine, Tel Aviv University, Tel Aviv, Israel; 14 Liver Disease Center, Sheba Medical Center, Ramat Gan, Israel

**Keywords:** Hepatitis delta (HDV), HDV genotype, Seroprevalence, Hepatitis B (HBV), HBV genotype, HBV/HDV viral load

## Abstract

**Background:**

Characteristics of hepatitis B (HBV) and delta (HDV) coinfection in various geographical regions, including Israel, remain unclear. Here we studied HDV seroprevalence in Israel, assessed HDV/HBV viral loads, circulating genotypes and hepatitis delta antigen (HDAg) conservation.

**Methods:**

Serological anti HDV IgG results from 8969 HBsAg positive individuals tested in 2010-2015 were retrospectively analyzed to determine HDV seroprevalence. In a cohort of HBV/HDV coinfected (*n*=58) and HBV monoinfected (*n*=27) patients, quantitative real-time PCR (qRT-PCR) and sequencing were performed to determine viral loads, genotypes and hepatitis delta antigen (HDAg) protein sequence.

**Results:**

6.5% (587/8969) of the HBsAg positive patients were positive for anti HDV antibodies. HDV viral load was >2 log copies/ml higher than HBV viral load in most of the coinfected patients with detectable HDV RNA (86%, 50/58). HDV genotype 1 was identified in all patients, most of whom did not express HBV. While 66.6% (4/6) of the HBV/HDV co-expressing patients carried HBV-D2 only 18.5% (5/27) of the HBV monoinfections had HBV-D2 (*p*=0.03). Higher genetic variability in the HDAg protein sequence was associated with higher HDV viral load.

**Conclusions:**

The overall significant prevalence of HDV (6.5%) mandates HDV RNA testing for all coinfected patients. Patients positive for HDV RNA (characterized by low HBV DNA blood levels) carried HDV genotype 1. Taken together, the significant HDV seroprevalence and the lack of effective anti-HDV therapy, necessitates strict clinical surveillance especially in patients with higher HDV viral loads and increased viral evolution.

## Background

More than 350 million people worldwide are chronically infected with hepatitis B virus (HBV). Fifteen to twenty millions of these individuals are considered to be coinfected with hepatitis delta virus (HDV) [1]. While HBV is a DNA virus coding for several proteins, HDV is a circular single-strand negative-sense ribonucleic acid (RNA) virus that codes for a single protein, the HDV antigen (HDAg) [2] and requires HBV surface antigen (HBsAg) for infection, thus can only be detected in HBV positive individuals. Infection with HBV and HDV leads to the most severe form of chronic viral hepatitis, causing liver cirrhosis and hepatocellular carcinoma [3]. Patients with HBV and HDV chronic infection have a twofold higher risk to develop cirrhosis, a threefold higher risk to develop hepatocellular carcinoma and a twofold increased mortality rate compared with HBV monoinfected individuals [4, 5]. The severity of the liver disease caused by HBV/HDV is thought to be associated with the HDV genotype and viral loads [6, 7]. HBV genome is divided into 10 genotypes (A-J). The most common HBV genotype in Europe is genotype D. There are eight reported genotypes of HDV with unexplained variations in their geographical distribution and pathogenicity [8]. The most common, worldwide distributed HDV genotype is genotype 1, found mainly in Europe and North America. While drugs for HBV are available and several anti-HDV drugs are now in clinical development [9, 10] there is still no specific therapy approved for HDV.

In Israel, the incidence of HBV has declined dramatically since the introduction of the vaccination program in 1992, and was estimated to be 0.5/100,000 in 2015 [11]. The prevalence of HBV was lately estimated to be 0.96% [12] suggesting that approximately 72,000 individuals in Israel are HBV carriers. On the other hand, the frequency of HBV/HDV infection has not been reported and information on the local genotypes which may be relevant when anti-HDV therapy will be available, is lacking.

Here, the seroprevalence of HDV was determined in a large sample of HBsAg positive patients. HBV and HDV viral loads and genotypes were assessed in a separate sample of patients positive for HDV RNA (*n*=58) and in 27 HBV monoinfected patients. In addition, the full HDAg coding region was determined and compared between HDV/HBV patients.

## Methods

### Patients and samples

Seroprevalence of HDV in Israel was determined by a retrospective analysis of anti HDV immunoglobulin G (IgG) antibody results obtained from all HBsAg positive patients (*n*=8969) tested for anti HDV between 2010 and 2015. The medical reason for requesting HDV serology by the physicians was not recorded.. The data was not obtained from a specific group of individuals (e.g. routine blood donors). The data was collected anonymously from approximately a quarter of the clinical virology laboratories engaged in HBV and HDV testing in Israel. These laboratories are located in five medical centers (Carmel, Soroka, Sheba, Rabin and Sorasky) from different regions in Israel. HDV IgG positivity was determined using an ELISA assay (ETI-AB-DELTAK-2, Dia-Sorin, Italy). All serological tests were performed following the manufacturer’s instructions. Whenever possible, information on sex and age was also collected. To reduce redundancy as much as possible, if multiple results from a single individual were identified, they were removed prior to data analysis. The presence of HDV-RNA, which was assessed by a qualitative assay [13] in HDV IgG positive samples identified in the clinical virology laboratory of Soroka, was also recorded. The study was approved by the Ethical Committee of the Sheba Medical Center (approval number SMC 2890-15) and informed consent was deemed unnecessary.

Molecular analysis (quantification of HDV and HBV viral load, genotyping and in HBV/HDV samples- comparison of HDAg predicted protein sequences) was performed on samples from 58 patients positive for HDV RNA and 27 HBV monoinfected patients (who failed anti HBV therapy and for whom HBV resistance analysis was requested). Blood samples (5 ml) collected from these patients between January 2013 and December 2016 were transferred to the national HIV and viral hepatitis reference center where plasma was separated and stored at -20^0^C until used.

### HDV and HBV viral load

Nucleic acids were extracted from 0.5 ml plasma using the NucliSENS Easy MAG total nucleic acid extraction system (Biomerieux, Marcy l'Etoile, France), according to the manufacturer's protocol. HDV viral load was determined with Primerdesign HDV genesig assay (Primerdesign Ltd, United Kingdom) which is characterized by high priming efficiencies of >95% and can detect less than 100 copies of target template and was validated with an external control program (QCMD HDV14, QCMD, Glasgow, Scotland). HBV viral load was determined as previously described [14]. This assay, with an estimated 20 IU/ml detection limit, was validated using an external control program (HBVDNA2017, QCMD, Glasgow, Scotland).

### HDV and HBV genotyping

Genotypes were determined in samples with viral load >1000 copies/ml (HBV or HDV). HDV genotype was determined following PCR amplification of the whole HDAg region with ORF891F 5’- ATGCCGACCCGAAGAGGA-3’ and ORF1680R 5’- GTCCAGCRGTCTCCTCTTTA-3’ by single step RT-PCR using 7 μl HDV RNA and sequencing with previously published primers [13, 15]. Genotyping of HBV was performed following PCR amplification of a fragment from the polymerase region with 2F-5-‘GCGGGCCGGCTACTCTTCTTTC and 6r 5’-GTGGGGGTTGCGTCAGCAAA-3’ with 5μl HBV DNA. Direct sequencing of all PCR products was performed using an automatic sequencer (ABI PRISM 3100 genetic analyzer DNA Sequencer, Applied Biosystems, Foster City, CA, USA) and BigDye Terminator v1.1 Cycle Sequencing kit (Applied Biosystems, Foster City, CA, USA).

### Phylogenetic analyses

HDV and HBV nucleotide sequences were aligned using the Open-gene system (Siemens, Malvern PA, United States). Reference sequences were GeneBank accession X04451 for HDV and the references sequences from Open-gene system module for HBV. Representative nucleotide sequences for HDV genotypes 1-8 from GenBank (Genotype 1a: U81989-Etiopia, U81988-Somalia; Genotype 1b: JX888099-Nigeria Genotype 1c: KJ744240-Iran; M58829-Nauru; Genotype1d: AM779588_tk34_Turkey; LT594475_Romania; KJ744214_Iran; KJ744218_Iran KR363258_1054_China; genotype2_AF104264; genotype3_L22063; genotype4_AF018077; genotype5_AM183326; genotype6_AM183332; genotype7_AM183333; genotype8_AM183327) and of HBV (D1_ AB674425,M32138, D2_EU594433, JF754621, D3_X85254, AB674437, A1_AF09084, FM199974, A2_GQ477460, X51970, C1_X04615,AB014381, C2_AB117758 , B3_AB033554, B1_D00329,B2_AF100309) were aligned using Sequencher 5.0, and clustered with the Clustal X algorithm (bootstrap value of 1000). Phylogenetic trees (375 nucleotides, 1200-1573 in HDV X04451 and 442 nucleotides, 458-897 in HBV AB674436) were constructed by neighbor joining method and produced by Mega 6.0.

### Comparison of HDAg protein sequences

Full length HDAg sequences were translated via online Expasy translation tool [16]. To investigate the mutational pattern of functional domains within the HDAg protein, the amino acid sequences of the different isolates were compared with each other using LOGO [17]. Shannon entropy was used to assess the degree of amino acid conservation [18]. Conserved residues are those with zero Shannon entropy.

### Statistical analysis

The association between HDV seropositivity, age and sex was assessed using the χ2 test for categorical variables and t-test for numeric variables. Univariate logistic regression model, Odds ratios (OR) and 95% confidence intervals were calculated. Fisher exact test was used to assess the association between HDV and HBV genotypes and between HDAg amino acid conservation in low (<5 log copies/ml) and high (>5 log copies/ml) HDV viral load patients. P-value <0.05 was considered statistically significant. Data were analyzed using the SAS software (version 9.1.3).

## Results

### HDV seroprevalence

Retrospective study of anti HDV IgG results obtained for samples from 8969 HBsAg positives was conducted, 587 (6.5%) were found positive (Table 1). Patients positive for anti IgG HDV were, on average, slightly older than HDV seronegative HBsAg positive patients (47.5±13.8 versus 45.2±16 years, *p*<0.01). No significant difference in HDV prevalence was found between males and females (6.2% versus 6.9%, respectively). The presence of HDV RNA was assessed in the Soroka virology laboratory for a limited number of samples found to be anti HDV positive (*n*=196). 23% (45/196) were HDV-RNA positive.

**Table 1:** Prevalence of HDV infection in Israel

	Samples tested, N	HDV negative samples	HDV seropositive samples	% seropositive (95% CI)	Odds Ratio (95% CI)	*p*-value
Total	8969	8382	587	6.5 (6.1-7.1)		
Age (mean± SD)	8452^a^	45.2± 16 (*n*=7919)	47.5± 13.8 (*n*=533)		1.0 (1.0-1.1)	<0.01
Gender (*n*=8744)^a^						
Male	5046	4734	312	6.2 (5.6-6.9)	Reference	0.18
Female	3698	3443	255	6.9 (61.-7.8)	1.1 (0.9-1.3)	

^a^The number of samples for which this information was available

### HDV and HBV viral load and genotypes

The mean and median HDV and HBV viral loads, assessed in a separate cohort of HBV/HDV patients (*n*=58) positive for HDV RNA, were 5.78±1.42 and 5.79 (IQR 2.3); 1.34±1.39 and 1.51 (IQR 1.58) log copies/ml, respectively, p<0.05. In most of these coinfection cases (86%, 50/58) HDV viral load was at least 2 log higher than HBV viral load. In samples from HBV monoinfected patients who failed anti HBV therapy (*n*=27), the mean and median HBV viral load was 4.8±1.66 and 4.6 (IQR 2.0) log copies/ml, respectively.

**Fig. 1:**
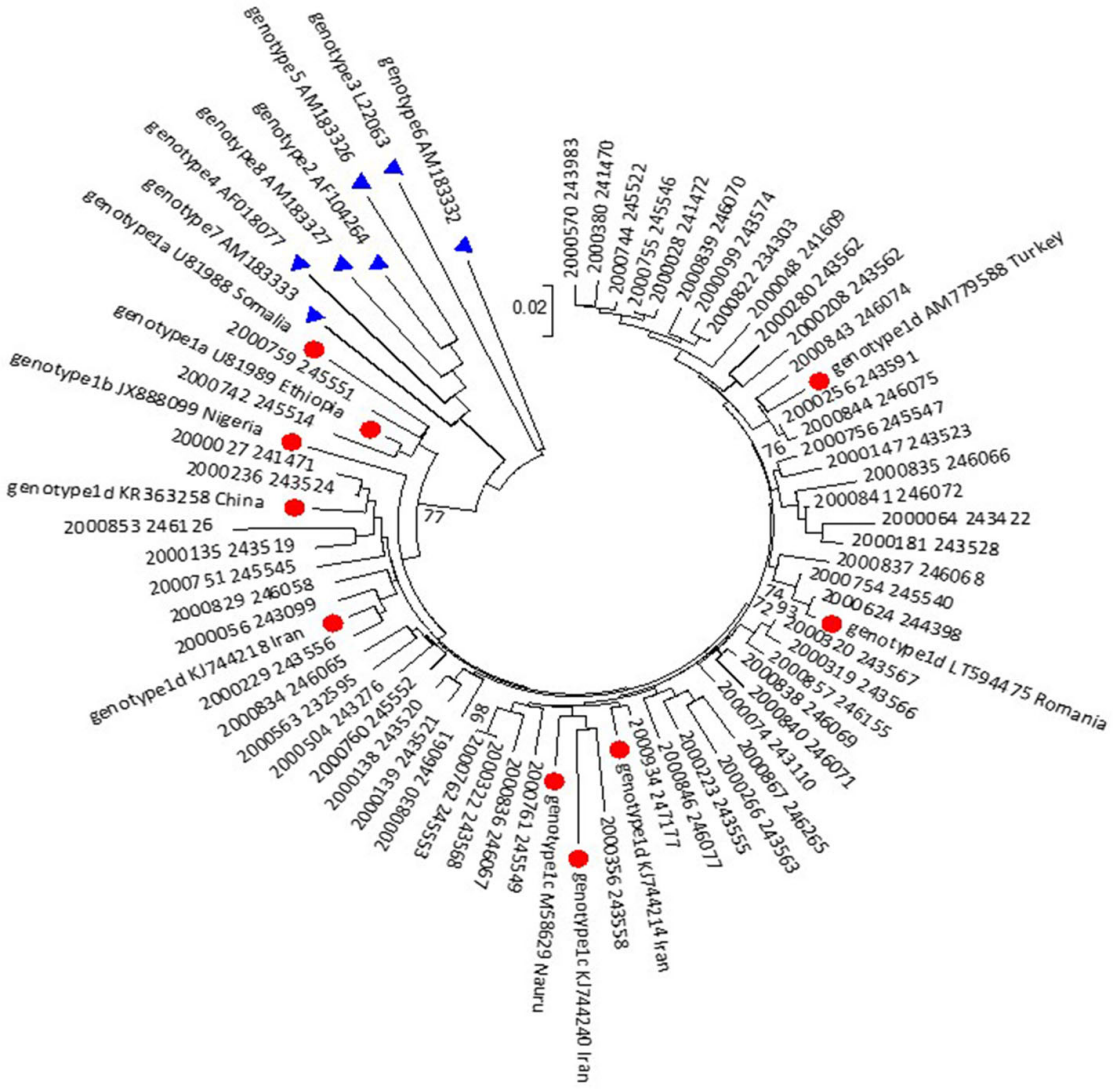
Phylogenetic Analysis of HDV nucleotide sequences. Phylogenetic tree of 55 HDV nucleic acid (n.a) patient sequences, 1 HDV-RNA sequence derived from HK293 cells transfected with HDV plasmid (2000504), and 17 prototype sequences obtained from GeneBank (dots, HDV genotype 1; triangle, HDV genotype 2-8) was constructed by Clustal W pairwise alignment with the 375 n.a. from HDVAg. The prototype sequences are identified by the HDV genotype and subtype [19], GeneBank accession number and the name of the country of origin (if known). The Israeli-identified sequences are defined by their patient and sample numbers. Bootstrap values are indicated for the major nodes as data obtained from 1000 replicates.

HDV genotype was successfully determined for 95% (55/58) of the HDV/HBV patients, all with >1000 copies/ml HDV viral load (Fig. 1). All were infected with HDV genotype 1. Le Gal et al., recently suggested classification of HDV-1 into four subtypes [19]. Accordingly, the majority (*n*=48) of the patients carried subtype HDV-1d and a small group (*n*=5) carried HDV-1c or HDV-1a (*n*=2). Of 55 samples in which HDV genotypes were determined, 41 had known birth place. While those from Eastern Europe (35/41, former Soviet Union, Ukraine, Romania) and Israel (2/41) carried HDV-1d or HDV-1c (2/41, Russia), both of the two patients born in Ethiopia carried HDV-1a which corresponds mainly to Africa [19, 20].

HBV genotype could be determined in samples from 33 patients: 6 HBV/HDV patients (those with plasma viral load >1000 copies/ml, enabling sequencing of both viruses) and in 27 HBV monoinfected patients.

Of 6 HBV/HDV patients, the distribution of HBV genotypes and subgenotypes was as below: 4 (66.7%), 1 (16.7%), and 1 (16.7%) were D2, D1 and D3 respectively. In contrast, of 27 HBV mono-infected patients, the distribution was as follow: D1 (59.3%), D2 (18.5%) D3 (11.1%) and A1, A2 and C2 (3.7% each). The prevalence of genotype HBV-D2 was significantly different between mono-infected and co-infected groups (4 (66.7%) vs 5 (18.5%), *p*=0.03).

### HDAg analysis

**Fig. 2:**
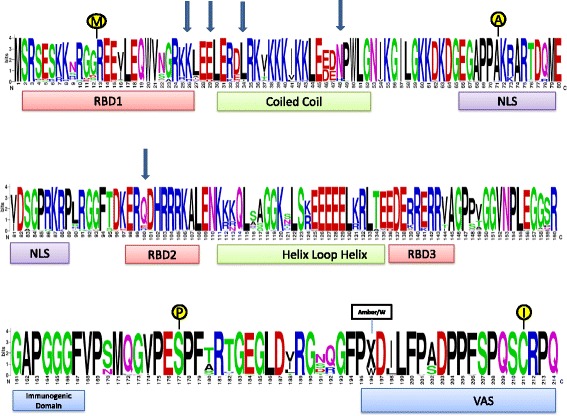
HDAg sequence alignment logo. Amino-acid logo of full HDAg sequences obtained from 48 HDV- RNA positive patients. Functional domains (presented by boxes) and post-transcriptional modification sites (illustrated as circles) are indicated. Arrows indicate residues in regulatory regions found to be less conserved in high viremic patients.

Full HDAg sequence (214 amino acids) was successfully determined in 48 of the 55 HBV/HDV patients with HDV sequences. It is possible that the considerable heterogeneity and strong internal base pairing of HDV did not allow analysis of the complete HDAg region in seven of the sampled [21]. Multiple sequence alignment of the full HDAg sequences revealed that although major regulatory sites [2, 22–28] were conserved in all patients, high amino-acid diversity was observed between the sequences. To better assess this phenomenon, the amino acid variability (as measured by Shannon Entropy) between 12 patients with low HDV viremia (<5log copies/ml, mean viral load 4.2±0.63 log copies/ml) and 36 with high HDV viremia (=>5 log copies/ml, mean viral load 6.6±1.42 log copies/ml) was compared. For this comparison, the same reference sequence was used (CAQ16911.1 large Hepatitis delta antigen dTk5 Turkey). In the 12 patients with low HDV viremia, the HDAg predicted protein sequence was more conserved (69.2%, 148/214 conserved residues) compared to the amino acid sequence of HDAg in the high viremic patients (56.1%, 120/214 conserved residues, *p*<0.05) versus. Substitutions in amino acids K26R, E29D, L34I/S/V, N58H/Y and Q100R/E located in the coiled coil and RNA binding domains were observed in high viremic patients only (Fig. 2).

## Discussion

HDV infection in patients with chronic HBV infection, which continues to be a public health concern worldwide, results in the most severe form of viral hepatitis. However, testing for hepatitis delta is limited and the rate of HDV infection in many countries is unknown [29]. To the best of our knowledge the rate of HDV seropositivity in Israel has not yet been reported nor have the circulating genotypes been assessed. Moreover, molecular detection of HDV by real-time PCR is rarely performed and HDV viral load is not monitored.

Here, 6.5% of HBsAg positive patients in Israel were found to be HDV seropositive, a higher rate than the estimated 5% worldwide prevalence [30]. Reported HDV prevalence varies in different European countries between very low (0.23%) in Slovenia to high (35.3%) prevalence in Greece [31]. In Lebanon HDV prevalence reported in 258 HBV positive individuals was only 1.2% [32]. A higher seroprevalence, 8.3%, was reported in Egypt among 121 HBV carriers [33]. As both studies assessed HDV seroprevalence in a small number of HBV seropositive individuals, the results may not fully represent the overall HDV infection rate in these countries. In a previous study performed in Israel on 400 injecting drug users, 22 were found HBsAg positive and 18% of them (4/22) were also anti HDV positive [34]. This high prevalence may be misleading as the study was performed on a small number of a specific group of individuals, before the initiation of HBV vaccination program and thus may not fully represent the current serological and demographic status of the population in Israel. The 6.5% rate for anti HDV antibodies found here amongst nearly 9000 HBsAg positives (>10% of the individuals considered to be HBV positive, [12]), may better present the current status of HDV infection in Israel. HDV RNA was detected in 24% of the samples tested for the presence of virus. These results, which may underestimate HDV RNA positivity due to low sensitivity of the assay and which were obtained in only a limited number of HDV seropositive patients, call for molecular analysis of HDV in all seropositive cases.

Genotyping revealed that HDV patients, most of whom were born in Eastern Europe, carried HDV-1 sequences which clustered with HDV isolates from Europe (Italy, Romania) and Asia (Iran, Turkey). The only two patients with HDV born in Ethiopia clustered with strains identified in Africa. Predominance of HDV-1 has been described worldwide [28, 35]. HDV-1 was also the only genotype identified in other Mediterranean countries like Turkey [36] and Tunisia [37].

Almost all (>90%) of HBV positive cases were infected with genotype D, the most prevalent genotype worldwide. However, while D1 was the most frequent subtype in HBV monoinfected patients (59.3%, 16/27) and D2 was identified in only 18.5% of these monoinfected patients, HBV/HDV patients were mainly (66.7%) infected with HBV-D2. HBV genotypes other than genotype D were not observed. This analysis was performed in a limited number of patients only, as most HBV/HDV coinfections had low or undetectable HBV viral load. Low viral loads of HBV in samples positive for plasma HDV RNA were previously reported by others [38], suggesting that HDV replication is associated with suppression of HBV replication [39]. Evidence has indicated that HDAg down regulates HBV replication by repressing activity of the two HBV enhancer regions, and by transactivating the interferon-inducible MxA gene, which inhibits HBV replication by reducing the export of viral mRNA from the nucleus [40]. In a study conducted in Spain which assessed viral loads of HDV in HBV-D carriers [7], higher HDV levels were significantly and persistently found compared to HBV. Specifically, this effect was HBV genotype dependent and was more pronounced in HBV-A than HBV-D genotypes (median of 4.48 and 3.49 log copies/ml for HBV-A and HBV-D, respectively). The authors concluded that higher inhibitory effect of HDV on HBV replication is HBV genotype specific. Our results show that subtype HBV-D2 was more frequently found in HBV/HDV coinfected individuals than in HBV monoinfected patients. It is possible that HBV-D2 overcomes the repression conferred by HDV-1 better than other HBV genotypes. The correlation between HDV and HBV genotypes and reciprocal repression has practical consequences. It may cause misdiagnoses of the viral load of HDV or HBV in coinfection and underrepresentation of slow replicating genotypes. Future studies are needed to better establish the association between HBV/HDV genotypes and co-replication interference.

HDAg protein is considered to display more amino acid changes compared to structural proteins of other RNA viruses [39]. Analysis of HDAg predicted amino acid sequences of 48 patients showed increased variability in high compared to low viremic patients in protein domains involved in viral replication. The functional and clinical relevance of the specific amino acid changes observed herein (including the changes in amino acid 202 observed in several patients not connected to viral load status) requires further studies especially as no direct acting HDV antiviral is yet available. Indeed, a major obstacle in developing treatment of HDV infection is lack of self-replicative function to be directly targeted by antivirals. Peg-Interferon remains the mainstay of treatment however interferon therapy is associated with frequent side effects and low response rate. Clinical studies exploring prenylation inhibitors, viral entry inhibitors and nucleic acid polymers to block particle release demonstrate progress towards cure of HDV infection [41].

One of the limitations of this study is that HDV-RNA measurements are not done routinely in all HDV seropositive cases, therefore, the rate of RNA positivity could be defined in only a limited number of cases. Also, HDV viral load was not routinely assessed. In addition, another limitation of this study is that data on disease status (e.g. cirrhosis), HBsAg levels, a known risk factor for HDV viremia [42] or risk factors for HDV infection (e.g. birth place, use of injection drugs) for the 586 seropositive HDV individuals was lacking. Although most HDV sequences in this study derived from patients born in Eastern European countries and a few in Ethiopia, countries with a high prevalence of HBV infection [43], this information was not available for all HDV seropositive individuals. The ethnic group of the HBV/HDV coninfected individuals, which may also be a risk factor for HBV positivity [42, 44] was also unknown.

## Conclusions

This study identified a 6.5% rate of HDV seroprevalence in HBsAg positive patients. This high rate suggests that screening for HDV in HBV positive patients in Israel is mandatory and should be continued. Furthermore, the presence of HDV-RNA should be assessed in all HDV IgG positive cases. In HDV-RNA positive cases, HDV viral load measurements could be beneficial. Patients with high HDV viral load and higher degree of viral diversity should be more closely monitored. Whenever possible, analysis of HBV/HDV genotypes will also be beneficial especially as the most common HDV genotype observed in this study, HDV-1, is considered to worsen the liver disease more than other HDV genotypes. Moreover, as the clinical efficacy of future anti-HDV therapy may also be influenced by HBV/HDV genotypes and subtypes, deciphering the local HBV/HDV status will be advantageous.

## Abbreviations

HBV: Hepatitis B
HDV: Hepatitis delta
HDAg: Hepatitis delta antigen
IgG: Immunoglobulin G
HIV: Human immunodeficiency virus
ml: Microliters
PCR: Polymerase chain reaction
RT-PCR: reverse transcription PCR
qRT-PCR: Quantitative real-time PCR

## References

[1] Wedemeyer H, Manns MP (2010). Epidemiology, pathogenesis and management of hepatitis D: update and challenges ahead. Nat Rev Gastroenterol Hepatol.

[2] Wang KS, Choo QL, Weiner AJ, Ou JH, Najarian RC, Thayer RM, Mullenbach GT, Denniston KJ, Gerin JL, Houghton M (1986). Structure, sequence and expression of the hepatitis delta (delta) viral genome. Nature.

[3] Lempp FA, Ni Y, Urban S (2016). Hepatitis delta virus: insights into a peculiar pathogen and novel treatment options. Nat Rev Gastroenterol Hepatol.

[4] Fattovich G, Bortolotti F, Donato F (2008). Natural history of chronic hepatitis B: special emphasis on disease progression and prognostic factors. J Hepatol.

[5] Fattovich G, Giustina G, Christensen E, Pantalena M, Zagni I, Realdi G, Schalm SW (2000). Influence of hepatitis delta virus infection on morbidity and mortality in compensated cirrhosis type B. The European Concerted Action on Viral Hepatitis (Eurohep). Gut.

[6] Braga WS, de Oliveira CM, de Araujo JR, Castilho Mda C, Rocha JM, Gimaque JB, Silva ML, Vasconcelos HL, Ramasawmy R, Parana R (2014). Chronic HDV/HBV co-infection: predictors of disease stage---a case series of HDV-3 patients. J Hepatol.

[7] Madejon A, Romero M, Hernandez A, Garcia-Sanchez A, Sanchez-Carrillo M, Olveira A, Garcia-Samaniego J (2016). Hepatitis B and D viruses replication interference: Influence of hepatitis B genotype. World J Gastroenterol.

[8] Le Gal F, Gault E, Ripault MP, Serpaggi J, Trinchet JC, Gordien E, Deny P (2006). Eighth major clade for hepatitis delta virus. Emerg Infect Dis.

[9] Koh C, Canini L, Dahari H, Zhao X, Uprichard SL, Haynes-Williams V, Winters MA, Subramanya G, Cooper SL, Pinto P, Wolff EF, Bishop R, Ai Thanda Han M, Cotler SJ, Kleiner DE, Keskin O, Idilman R, Yurdaydin C, Glenn JS, Heller T (2015). Oral prenylation inhibition with lonafarnib in chronic hepatitis D infection: a proof-of-concept randomised, double-blind, placebo-controlled phase 2A trial. Lancet Infect Dis.

[10] Bogomolov P, Alexandrov A, Voronkova N, Macievich M, Kokina K, Petrachenkova M, Lehr T, Lempp FA, Wedemeyer H, Haag M, Schwab M, Haefeli WE, Blank A, Urban S (2016). Treatment of chronic hepatitis D with the entry inhibitor myrcludex B: First results of a phase Ib/IIa study. J Hepatol.

[11] Farias A, Re V, Mengarelli S, Kremer L, Pisano MB, Allende L, Nicolas J, Elbarcha O, Contigiani M (2010). Detection of hepatitis C virus (HCV) in body fluids from HCV monoinfected and HCV/HIV coinfected patients. Hepatogastroenterology.

[12] Schweitzer A, Horn J, Mikolajczyk RT, Krause G, Ott JJ (2015). Estimations of worldwide prevalence of chronic hepatitis B virus infection: a systematic review of data published between 1965 and 2013. Lancet.

[13] Mederacke I, Bremer B, Heidrich B, Kirschner J, Deterding K, Bock T, Wursthorn K, Manns MP, Wedemeyer H (2010). Establishment of a novel quantitative hepatitis D virus (HDV) RNA assay using the Cobas TaqMan platform to study HDV RNA kinetics. J Clin Microbiol.

[14] Mixson-Hayden T, Lee D, Ganova-Raeva L, Drobeniuc J, Stauffer WM, Teshale E, Kamili S (2014). Hepatitis B virus and hepatitis C virus infections in United States-bound refugees from Asia and Africa. Am J Trop Med Hyg.

[15] Celik I, Karatayli E, Cevik E, Kabakci SG, Karatayli SC, Dinc B, Cinar K, Yalcin K, Idilman R, Yurdaydin C, Bozdayi AM (2011). Complete genome sequences and phylogenetic analysis of hepatitis delta viruses isolated from nine Turkish patients. Arch Virol.

[16] Artimo P, Jonnalagedda M, Arnold K, Baratin D, Csardi G, de Castro E, Duvaud S, Flegel V, Fortier A, Gasteiger E, Grosdidier A, Hernandez C, Ioannidis V, Kuznetsov D, Liechti R, Moretti S, Mostaguir K, Redaschi N, Rossier G, Xenarios I, Stockinger H (2012). ExPASy: SIB bioinformatics resource portal. Nucleic Acids Res.

[17] Crooks GE, Hon G, Chandonia JM, Brenner SE (2004). WebLogo: a sequence logo generator. Genome Res.

[18] Yusim K, Richardson R, Tao N, Dalwani A, Agrawal A, Szinger J, Funkhouser R, Korber B, Kuiken C (2005). Los alamos hepatitis C immunology database. Appl Bioinformatics.

[19] Le Gal F, Brichler S, Drugan T, Alloui C, Roulot D, Pawlotsky JM, Deny P, Gordien E (2017). Genetic diversity and worldwide distribution of the deltavirus genus: A study of 2,152 clinical strains. Hepatology.

[20] Zhang YY, Tsega E, Hansson BG (1996). Phylogenetic analysis of hepatitis D viruses indicating a new genotype I subgroup among African isolates. J Clin Microbiol.

[21] Homs M, Giersch K, Blasi M, Lutgehetmann M, Buti M, Esteban R, Dandri M, Rodriguez-Frias F (2014). Relevance of a full-length genomic RNA standard and a thermal-shock step for optimal hepatitis delta virus quantification. J Clin Microbiol.

[22] Alves C, Freitas N, Cunha C (2008). Characterization of the nuclear localization signal of the hepatitis delta virus antigen. Virology.

[23] Poisson F, Roingeard P, Baillou A, Dubois F, Bonelli F, Calogero RA, Goudeau A (1993). Characterization of RNA-binding domains of hepatitis delta antigen. J Gen Virol.

[24] Alves C, Branco C, Cunha C (2013). Hepatitis delta virus: a peculiar virus. Adv Virol.

[25] Mu JJ, Chen DS, Chen PJ (2001). The conserved serine 177 in the delta antigen of hepatitis delta virus is one putative phosphorylation site and is required for efficient viral RNA replication. J Virol.

[26] Glenn JS, Watson JA, Havel CM, White JM (1992). Identification of a prenylation site in delta virus large antigen. Science.

[27] Shih HH, Shih C, Wang HW, Su CW, Sheen IJ, Wu JC (2010). Pro-205 of large hepatitis delta antigen and Pro-62 of major hepatitis B surface antigen influence the assembly of different genotypes of hepatitis D virus. J Gen Virol.

[28] Perveen S, Nasir MI, Shahid SM, Azhar A, Khan OY (2012). Phylogenetic analysis of HDV isolates from HBsAg positive patients in Karachi, Pakistan. Virol J.

[29] Eduardo B. Martins JG (2017) Sa1486 – Prevalence of Hepatitis Delta Virus (HDV) Infection in the United States: Results from an ICD-10 Review. Gastroenterology Volume 152, Issue 5, Supplement 1, April 2017, Pages S1085

[30] Meng XJ (2010). Recent advances in Hepatitis E virus. J Viral Hepat.

[31] Jelen MM, Hosnjak L, Stunf S, Zagozen A, Fujs Komlos K, Markocic P, Poljak M, Seme K (2016). Hepatitis D virus infection in Slovenian patients with chronic hepatitis B virus infection: a national prevalence study and literature review. Acta Dermatovenerol Alp Pannonica Adriat.

[32] Ramia S, El-Zaatari M, Sharara AI, Ramlawi F, Farhat B (2007). Current prevalence of hepatitis delta virus (HDV) infection and the range of HDV genotypes in Lebanon. Epidemiol Infect.

[33] Fouad R, Abdo M, Eldeen HG, Sabry D, Atef M, Ahmed R, Zayed N (2016). Influence of delta virus infection on the virologic status in Egyptian patients with chronic hepatitis B virus genotype D. J Med Virol.

[34] Dan M, Rock M, Lilos P, Shany SB (1993). Seroepidemiology of hepatitis B and D virus infection among intravenous drug addicts in Israel. Int J Epidemiol.

[35] El Bouzidi K, Elamin W, Kranzer K, Irish DN, Ferns B, Kennedy P, Rosenberg W, Dusheiko G, Sabin CA, Smith BC, Nastouli E (2015). Hepatitis delta virus testing, epidemiology and management: a multicentre cross-sectional study of patients in London. J Clin Virol.

[36] Altuglu I, Ozacar T, Sertoz RY, Erensoy S (2007). Hepatitis delta virus (HDV) genotypes in patients with chronic hepatitis: molecular epidemiology of HDV in Turkey. Int J Infect Dis.

[37] Yacoubi L, Brichler S, Mansour W, Le Gal F, Hammami W, Sadraoui A, Ben Mami N, Msaddek A, Cheikh I, Triki H, Gordien E (2015). Molecular epidemiology of hepatitis B and Delta virus strains that spread in the Mediterranean North East Coast of Tunisia. J Clin Virol.

[38] Gish RG, Yi DH, Kane S, Clark M, Mangahas M, Baqai S, Winters MA, Proudfoot J, Glenn JS (2013). Coinfection with hepatitis B and D: epidemiology, prevalence and disease in patients in Northern California. J Gastroenterol Hepatol.

[39] Shirvani-Dastgerdi E, Tacke F (2015). Molecular interactions between hepatitis B virus and delta virus. World J Virol.

[40] Alfaiate D, Lucifora J, Abeywickrama-Samarakoon N, Michelet M, Testoni B, Cortay JC, Sureau C, Zoulim F, Deny P, Durantel D (2016). HDV RNA replication is associated with HBV repression and interferon-stimulated genes induction in super-infected hepatocytes. Antiviral Res.

[41] Wranke A, Wedemeyer H (2016). Antiviral therapy of hepatitis delta virus infection - progress and challenges towards cure. Curr Opin Virol.

[42] Lin HH, Lee SS, Yu ML, Chang TT, Su CW, Hu BS, Chen YS, Huang CK, Lai CH, Lin JN, Wu JC (2015). Changing hepatitis D virus epidemiology in a hepatitis B virus endemic area with a national vaccination program. Hepatology.

[43] Loebstein R, Mahagna R, Maor Y, Kurnik D, Elbaz E, Halkin H, Olchovsky D, Ezra D, Almog S (2008). Hepatitis C, B, and human immunodeficiency virus infections in illicit drug users in Israel: prevalence and risk factors. Isr Med Assoc J.

[44] Sandler SG, Nath N, Biger Y (1977). Seroepidemiology of hepatitis B virus in Israel. Results of a pilot study in Jerusalem. Am J Epidemiol.

